# Monitoring the Growth of an Orthotopic Tumour Xenograft Model: Multi-Modal Imaging Assessment with Benchtop MRI (1T), High-Field MRI (9.4T), Ultrasound and Bioluminescence

**DOI:** 10.1371/journal.pone.0156162

**Published:** 2016-05-25

**Authors:** Rajiv Ramasawmy, S. Peter Johnson, Thomas A. Roberts, Daniel J. Stuckey, Anna L. David, R. Barbara Pedley, Mark F. Lythgoe, Bernard Siow, Simon Walker-Samuel

**Affiliations:** 1 UCL Centre for Advanced Biomedical Imaging, Division of Medicine, London, United Kingdom; 2 UCL Cancer Institute, London, United Kingdom; 3 UCL Institute for Women’s Health, London, United Kingdom; Northwestern University Feinberg School of Medicine, UNITED STATES

## Abstract

**Background:**

Research using orthotopic and transgenic models of cancer requires imaging methods to non-invasively quantify tumour burden. As the choice of appropriate imaging modality is wide-ranging, this study aimed to compare low-field (1T) magnetic resonance imaging (MRI), a novel and relatively low-cost system, against established preclinical techniques: bioluminescence imaging (BLI), ultrasound imaging (US), and high-field (9.4T) MRI.

**Methods:**

A model of colorectal metastasis to the liver was established in eight mice, which were imaged with each modality over four weeks post-implantation. Tumour burden was assessed from manually segmented regions.

**Results:**

All four imaging systems provided sufficient contrast to detect tumours in all of the mice after two weeks. No significant difference was detected between tumour doubling times estimated by low-field MRI, ultrasound imaging or high-field MRI. A strong correlation was measured between high-field MRI estimates of tumour burden and all the other modalities (p < 0.001, Pearson).

**Conclusion:**

These results suggest that both low-field MRI and ultrasound imaging are accurate modalities for characterising the growth of preclinical tumour models.

## Introduction

Translation of research in oncology, from basic biology to therapeutic drug strategies, requires the use of experimental models of disease. Transgenic and orthotopic models are increasingly used to study a tumour microenvironment that more accurately reflects the growth and clinical presentation of the disease [1] than in traditional subcutaneous tumour models [2–4]. Whilst these models have the potential to improve the translation of novel therapeutic strategies, monitoring tumour growth and response to therapy is generally not straightforward. In particular, transgenic models develop tumours spontaneously and over a longer period compared to implanted orthotopic models, hence a screening method is vital. Tumour sites can be inaccessible to traditional calliper measurement of tumour volume [5], and invasive methods used to assess tumour volume require the termination of entire animal groups, which is incompatible with longitudinal studies and greatly increases the number of animals required. Biomedical imaging is key to overcoming this limitation [6], and, additionally, facilitates paired statistical analysis from longitudinal measurements and reduces the required cohort sizes. However, which imaging modality offers the best and most practical solution is often difficult to determine.

In recent years, small-animal imaging has rapidly evolved and many dedicated platforms exist [7], including magnetic resonance imaging (MRI), ultrasound imaging (US), x-ray computed tomography (CT), positron emission tomography (PET), single photon emission computed tomography (SPECT), fluorescence imaging (FLI), photoacoustic imaging (PAI) and bioluminescence imaging (BLI). More recently, low-field, ‘benchtop’ MRI scanners have been developed, which offer promise for relative low-cost and precise imaging of pre-clinical models [8–11]. Several of these imaging modalities can also provide functional information on tumour function and microstructure, such as blood flow [12;13], cell density [14], hypoxia [15] and metabolite concentration [16], each of which has been used to assess response to anti-cancer therapy. However, particularly for drug development in cancer, the parameter of greatest interest still tends to be tumour volume. For translational research, measurement of the change in tumour volume allows direct comparison with the clinical assessment via the RECIST criteria [17]. Determining pre-therapy tumour volumes of animal models in therapy trials is essential for group standardisation, alongside the confirmation of successful engraftment.

Measuring tumour volume is generally straightforward and fast, although the imaging modality that provides the optimal platform, both in terms of scientific and practical considerations, depends on the requirements of a particular study. The accessibility of each small animal imaging platform will vary between institutions and research groups, and deciding which systems to use can be complex, and has led to a few studies comparing the application of different systems [7;18]. However, only a limited number of studies have directly compared small-animal MRI and US imaging systems in assessing tumour volumes, though there are some alternative applications and clinical reports available [19–22]. A key advantage of non-invasive imaging is its ability to monitor individual animals longitudinally [23;24]. However, the efficacy of novel ‘benchtop’ MRI is currently unreported in this context.

The aim of this study was to assess the ability of a low-field MRI system (field strength 1T) to longitudinally characterise and quantify the growth of a mouse liver metastatic model whilst directly comparing its performance to three small-animal imaging platforms: high-field MRI (field strength 9.4T); bioluminescence imaging (BLI); and ultrasound imaging (US). Of all the available modalities, these provide the best potential for assessing tumour burden, without the use of ionising radiation. We hope that this study may provide useful information for imaging laboratories planning tumour growth characterisation studies or looking to invest in new imaging equipment.

### Imaging Systems Overview

Preclinical MRI is generally performed using high-field systems (4.7T – 11.75T), due to the signal-to-noise gains that can be achieved with larger magnetic fields. Such imaging systems are able to characterise both anatomical and functional information across the whole body. However, high-field systems are expensive, occupy a relatively large area and require routine maintenance due to the cryogenic cooling system of the superconducting magnet. Given the ability of MRI to allow precise and accurate quantification of tumour volume [25], it was considered the gold-standard for comparison with other techniques in this study.

Recently developed, low-field, permanent-magnet MRI systems have a number of advantages over high field systems such as their relative small footprint [8], and can be situated in standard biomedical research laboratories. Though the design is compact, it is suitable for *in vivo* pre-clinical research with mice and small rats. Such low-field MRI scanners inherently have lower signal-to-noise characteristics than their high-field counterparts, although the lower field can also lead to reduced image artefacts [26]. Moreover, shortened longitudinal relaxation times can reduce image acquisition time and provide greater image contrast [27;28]. Thus, high-field and low-field MRI were treated as different imaging modalities for the purpose of this study.

BLI is one of the most extensively used technologies for assessing biological function in preclinical models of disease [29;30]. The underlying mechanism of BLI is the enzymatic conversion of luciferin, resulting in the emission of light. The introduction of luciferase genes into non-expressing cells allows various biological functions to be assessed by the administration of a luciferin substrate. However, the resultant emitted light is of low intensity, and the scattering of the light in biological tissue makes quantification difficult. BLI is fast and highly sensitive, allowing very small numbers (<1000 [31]) of engineered cells to be detected *in vivo*. As such it is particularly suited to cell tracking, either of cancerous cells or cells of the immune system, in a longitudinal, non-invasive manner [32;33]. BLI systems are relatively compact, requiring a dark box and a sensitive camera for 2D imaging of the emitted light.

US imaging has been employed in preclinical research for several decades. Recent development of dedicated US systems with ultra-high frequency transducers (25–70 MHz) has revolutionised preclinical US imaging, allowing high temporal (<1 ms) and spatial (<30 um) resolution images of heart [34], blood flow, tumour volume [35;36], and embryonic development [37,38], each of which can be acquired in real-time and in three dimensions. However, US imaging is liable to inter-operator variability, and has limited application to imaging tumours propagated within the brain or bone due to signal attenuation. Preclinical ultrasound imaging systems are compact, and use a dedicated animal platform for ease of use with the transducer and stepping motor for 3D imaging. These developments mean that preclinical US may be a viable alternative to other imaging methods, due to its speed, cost effectiveness and relative ease of image acquisition.

## Methods

### Ethics statement

All animal studies were approved by the University College London Biological Services Ethical Review Committee and licensed under the UK Home Office regulations and the Guidance for the Operation of Animals (Scientific Procedures) Act 1986 (Home Office, London, United Kingdom) and United Kingdom Co-ordinating Committee on Cancer Research Guidelines for the Welfare and Use of Animals in Cancer Research [39].

### Cell line and orthotopic liver tumour xenograft

The human colorectal carcinoma cell line SW1222 was previously engineered in-house to express the luciferase gene [40]. A model of liver metastases, previously established in-house, was created in eight female MF1 *nu*/*nu* mice (6–8 weeks old, 25–30 g); defined as day 1. 1 x10^6^ SW1222 *luc* cells were injected intrasplenically in a Dulbecco’s Modified Eagle’s Medium vehicle (Sigma Aldrich, UK) via sterile surgical procedure so tumour cells washed through to the liver, where they established solid tumour deposits. Following splenectomy, to remove the possibility of tumours growing at the spleen, the laparotomy rectus sheath incision was sutured (Ethicon, Johnson & Johnson, USA) and the skin was closed with wound clips (Autoclip system, Harvard Apparatus, UK).

The mice were then monitored for development of solid tumour deposits via bioluminescence imaging, ultrasound imaging, low-field (1T) MRI and high-field (9.4T) MRI. Mice were allowed to recover for 24 hours between imaging sessions, in accordance with local regulations. US and BLI imaging were performed on the same day where possible; due to the proximity and efficiency of the systems, mice were kept anesthetised between the acquisitions. BLI and US imaging started at day 2. As the tumours were not expected to grow to a size detectable by the resolution of the high- and low-field MRI in the first week, these modalities began from day 7.

For all imaging, mice were anesthetised with 4% isoflurane in 1 L/min O_2_, and then maintained at 1.5% isoflurane using nose-cones within the imaging systems. Animals were monitored daily, mice that presented a tumour burden greater than 50% of the total liver volume were sacrificed by cervical dislocation, in accordance with the local regulations.

### Bioluminescence Imaging

BLI was performed on days 2, 9, 20 and 23 using a Photon Imager Optima (Biospace Lab, France). For whole body *in vivo* images, animals were administered 150 μL D-luciferin (Biosynth, USA) intraperitoneally at 150 mg/kg. Four mice were imaged simultaneously per acquisition. Peak emission had previously been determined to be at approximately 15 minutes post-injection, so animals were anaesthetised at 12 minutes and placed within the imaging chamber. Images were acquired in a single, supine orientation, with a 10 s exposure time.

### High-field (9.4T) MRI

High-field imaging was carried out on days 8, 14, 20 and 27 on an Agilent 9.4T horizontal-bore VNMRS scanner (Agilent Technologies, Santa Clara, USA) using a 39 mm diameter birdcage coil (RAPID Biomed, Rimpar, Germany). Animals were placed supine within a custom-built cradle. Core temperature was monitored with a rectal temperature probe, and maintained using heated water pipes. Respiratory rate was monitored using respiratory bellows, which were also used to gate the image acquisition (SA Instruments, NY, USA). Animals were then positioned within the scanner and a gated, manual shim was carried out over the entire liver volume.

Entire liver volumes were acquired with a respiratory triggered, multi-slice fast spin-echo sequence, typically using 30–35 slices. Sequence parameters included: in-plane resolution 0.156 x 0.156 mm^2^; slice thickness, 0.75 mm; effective echo time (TE), 19 ms; repetition time (TR), 1 s; 2 averages; total acquisition time was 9 minutes. These parameter values were chosen on the basis of previous in-house measurements of tumour and liver longitudinal (*T*_1_) and transverse (*T*_2_) relaxation times at 9.4T (Liver *T*_1_ = 1345 ± 280 ms and *T*_2_ = 18 ± 6 ms; tumour *T*_1_ = 2236 ± 544 ms and *T*_2_ = 35 ± 8 ms), which were used to numerically optimise the contrast between each tissue type [41]. These relaxation times are in good agreement with previous excised tissue measurements in colon carcinoma metastases at 9.4T [42].

### Benchtop, low-field (1T) MRI

Low-field MRI was performed on days 7, 15, 18, 23 and 28 on a Bruker 1T ICON (Bruker BioSciences Corporation, Ettlingen, Germany) using a 30 mm diameter solenoid coil. As mentioned above, the practical differences between low- and high-field MRI required that they were treated as different imaging modalities. However, the image resolution of low-field MRI was matched to those acquired on the high-field MRI scanner, and scan duration was limited to below 30 minutes. Animals were anesthetised and positioned prone within a holding cradle: physiological monitoring was provided via a rectal thermometer and respiratory bellows (SA Instruments, New York, USA), the temperature was maintained via a water-heated cradle. Once the animal was positioned within the scanner, automated power calibration and shimming were performed prior to pilot imaging.

Whole liver anatomical data were acquired using a respiratory-triggered, multi-slice fast spin echo. The datasets were resolution-matched to the high-field data, and acquired with the following parameters: effective TE, 25 ms; TR, 2s; 3 averages; total acquisition time, 25 minutes. As with the acquisition at 9.4T, these parameter values were chosen on the basis of a numerical optimisation that used the following relaxation times, which were characterised previously in-house: liver *T*_1_ = 310 ± 87 ms, *T*_2_ = 56 ± 34 ms; tumour *T*_1_ = 505 ± 278 ms, *T*_2_ = 78 ± 28 ms [41].

### Ultrasound Imaging

US imaging was performed on days 2, 9, 16 and 23 with a Vevo 2100 system (VisualSonics, Toronto, Canada) with an MS550D transducer. Mice were anaesthetised and placed supine on the handling table, where ECG and respiration were monitored using conductive strips built in to the imaging platform. The temperature was maintained using a heating lamp and monitored using a rectal probe. The abdomen of each mouse was covered with warmed acoustic coupling jelly. The transducer was placed in a sagittal orientation and a stepper motor was used to acquire a three-dimensional data set (resolution of 0.018 x 0.018 x 0.076 mm^3^) across the whole liver, using respiratory gating; total scan time of approximately 5 minutes.

### Image Segmentation

The mean photon flux from the BLI data was calculated over an automated region of interest (ROI) using M3Vision software (Biospace Lab, France). All data sets acquired with US, low-field MRI and high-field MRI were manually segmented into tumour and normal liver tissue by two researchers with at least four years of preclinical imaging experience, using Amira 5.4 (FEI, Oregon, USA). Segmentation was performed blind, following randomisation of the day and mouse number.

Whole liver volumes from low- and high-field MRI data were analysed. However, whole liver analysis wasn’t practically feasible for US images: the three-dimensional acquisition provided a very fine step thickness, and sagittal coverage of the whole liver typically yielded in excess of 250 slices. To enable segmentation within a reasonable timeframe, the data were compressed such that every three slices were averaged into one to yield a final slice thickness of 0.228 mm. In order to match the number of slices segmented by MRI, three representative samples of ten consecutive slices were chosen by each user, to account for heterogeneity of tumour engraftment. The relative tumour burden was resultantly used to compare the MRI and US for analysis of model development, calculated as the ratio of observed tumour to observed liver volume:

$$tumour\;burden=\;\frac{tumour\;volume}{liver\;volume}\times 100\%.$$ Equation 1

### Quantitative Comparison of Modalities

#### Tumour doubling time

Doubling times for tumour growth were estimated by fitting an exponential function of the form *V* = *V*_0_ exp(*Rt*) to estimates of tumour burden *V* (from Eq 1), as a function of time *t*. A linear-least squares algorithm in MATLAB (MathWorks, Natick, MA, USA) was used to estimate the rate constant *R*, and the doubling time *τ* was then given by *τ* = ln(2)/*R*. Tumour burden and flux were fitted for each mouse and for each modality, using data from day 9 to day 23, thus the same group size (n = 8) was maintained for all modalities. Day 2 BLI data was excluded from this analysis, as the signal included non-engrafted tumour cells.

#### Variability of intra-user and inter-user tumour burden assessment

The coefficient of variability was used to assess intra- and inter-user variability of segmentation for the US, low- and high-field MRI:

$$CV=\frac{\sigma }{\mu }\times 100\%,$$ Equation 2

Where σ is the standard deviation and μ is the mean. Five duplicate datasets were included within both user’s blinded segmentation, allowing intra-user variability (*CV*_U_) and inter-user variability (*CV*_A_) to be calculated. *CV*_U_ measures the user’s repeatability in assessing the data, and the *CV*_A_ gives the estimate of agreement between user segmentation of matching data. A separate dataset was performed blind in a given region to remove the possibility of user choice of analysed slices contributing to the variation in the US data.

#### Contrast to noise ratio estimation

The ability to discern tumour from normal liver is dependent on the difference in signal from the two tissue types and how this compares to the noise variance inherent in the data. The contrast-to-noise ratio (CNR) for MRI and US was calculated by defining two regions corresponding to tumour or normal-appearing liver. The mean (*μ*_L_, *μ*_T_) and standard deviation (*σ*_L_, *σ*_T_) of the signal within each region were then used in the following equation:

$$CNR=\frac{\left|\mu_{L}-\mu_{T}\right|}{\sqrt{\sigma_{L}{}^{2}-\sigma_{T}{}^{2}}}.$$ Equation 3

For an approximation of the signal-to-noise of the BLI, the mean signal of each tumour was normalised by the mean signal of a noise ROI drawn distal from the mice. As the mice were imaged in two sets of four, an ROI was drawn for each image at every week. The SNR was then calculated for the entire cohort over all the imaging time points.

#### Sensitivity calculation

A sensitivity measure was used to quantify the ability of each modality to detect tumours within the liver, compared with high-field MRI, taken as the gold-standard. Data sets from all time points and from each modality were denoted as either exhibiting tumours, or devoid of tumours. Sensitivity (*S*) at weeks 2–4, for low-field MRI and US, was calculated using: *S* = *TP*(*TP* + *FN*), using the high-field MRI data to determine true positives (TP) and false negatives (FN).

#### Statistical analysis

All statistical analysis was performed using MATLAB (MathWorks, USA). ANOVA was used in order to compare group-wise doubling times, choosing time points which ensure a consistent group size of eight. Pearson’s linear correlation was used to compare between the modalities estimation of tumour burden within the smallest period between imaging, omitting 0% burden values from all correlation calculations: high-field MRI (day 8, day 20) vs. BLI (day 9, day 20), high-field MRI (day 8, day 14, day 20, day 27) vs. low-field MRI (day 7, day 15, day 21, day 28), high-field MRI (day 8, day 14, day 20) vs. US (day 9, day 16, day 23).

## Results

Three mice were removed from the study at day 24 due to excessive tumour burden. All other mice (n = 5) remained in the study until the final time point at day 28.

**Fig 1:**
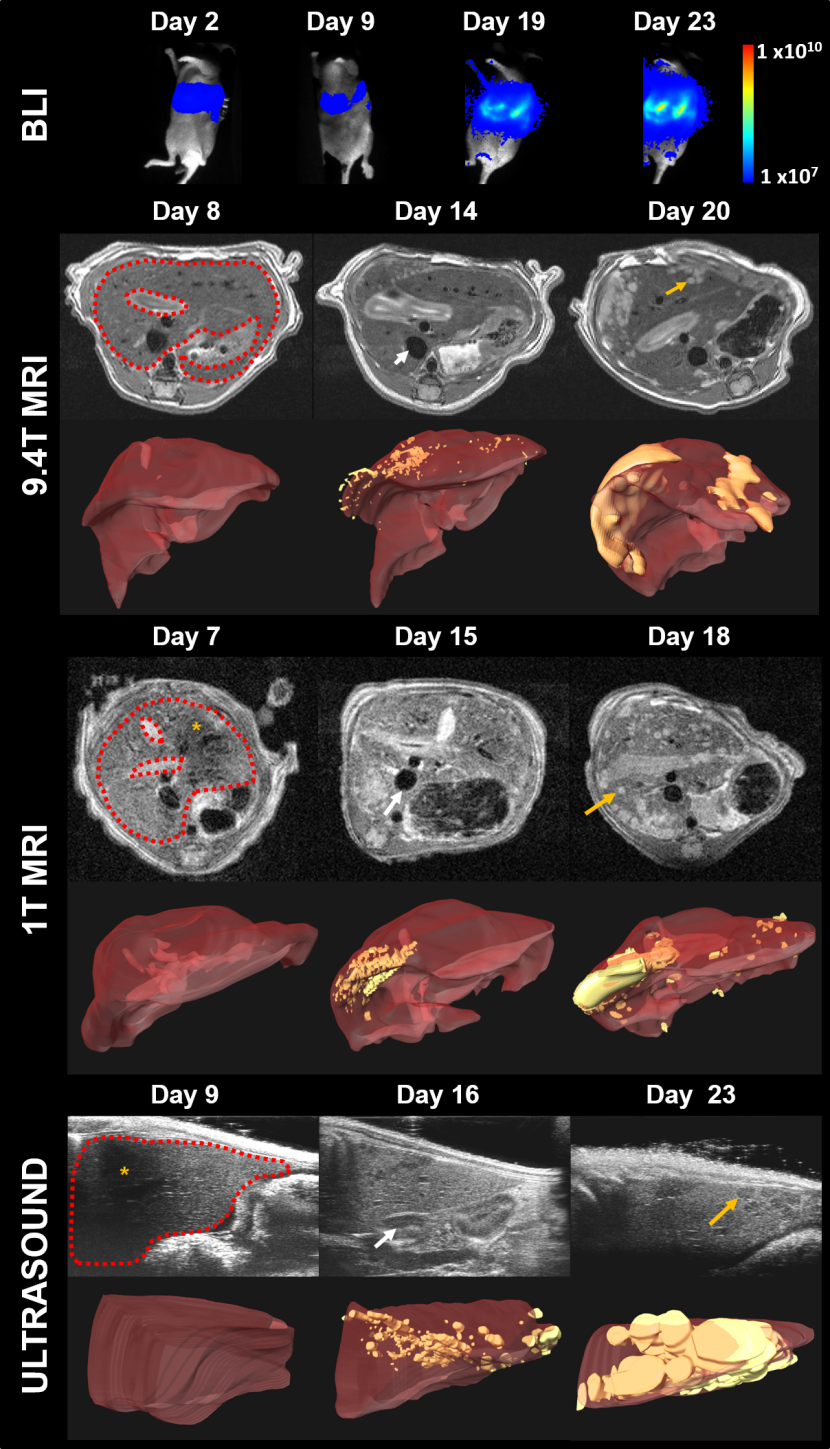
Representative images of the liver in a mouse model of colorectal metastasis, acquired over all time points, for all four modalities. Bioluminescence images (photon flux measured in p/s/cm^2^/str) have been scaled by the same logarithmic range across all time points. Bioluminescent signal can be seen at the location of the liver, which increases with the onset of the tumours. For US, high- and low-field MRI, typical slices are shown above corresponding three-dimensional visualisations of a manually segmented liver (red) and tumours (yellow). In the raw images, liver tissue has been delineated by a dashed red line, example tumours have been marked with orange arrows and example blood vessels are shown with a white arrow. Image artefacts have been demarcated with an orange star; signal loss occurs in MRI data due to cardiac motion, and ultrasound images suffer from transmission loss due to the ribs.

Fig 1 shows example images of the development of liver metastases in a mouse from BLI, high-field MRI, low-field MRI and ultrasound imaging with the corresponding three-dimensional visualisations over three weeks of imaging. BLI signal was detected in the region of the liver at day 2 in all mice (n = 8), and in the same region at all further time points, demonstrating successful engraftment of the liver tumour model in all mice. Due to the dynamic range over the weeks, the background signal in the final time-point has been amplified such that the BLI signal appears clipped at the location of the partitions between the mice when imaging multiple animals.

For the US, high and low-field MRI, the liver has been outlined (red dash), example tumours have been demarcated by an orange arrow, and example vasculature has been delineated with a white arrow. In the 3D visualisations, the liver volume has been rendered in red and the segmented tumours as yellow. Liver metastases increased in volume with time in all mice, starting as small, localised deposits often located at the periphery of the liver.

In the high-field (9.4T) MRI images, tumours appeared bright relative to the normal liver and blood vessels were dark. Images from low-field (1T) MRI were similar to high-field images, but with observably lower signal-to-noise characteristics, although tumours could still be discerned from liver. Some artefacts due to respiratory motion were evident (star), as shown in Fig 1. In US images, tumours appear hypo-intense relative to liver tissue and vasculature more so. Transmission shadowing caused by the ribs (star), was found to obscure some regions of the liver.

**Fig 2:**
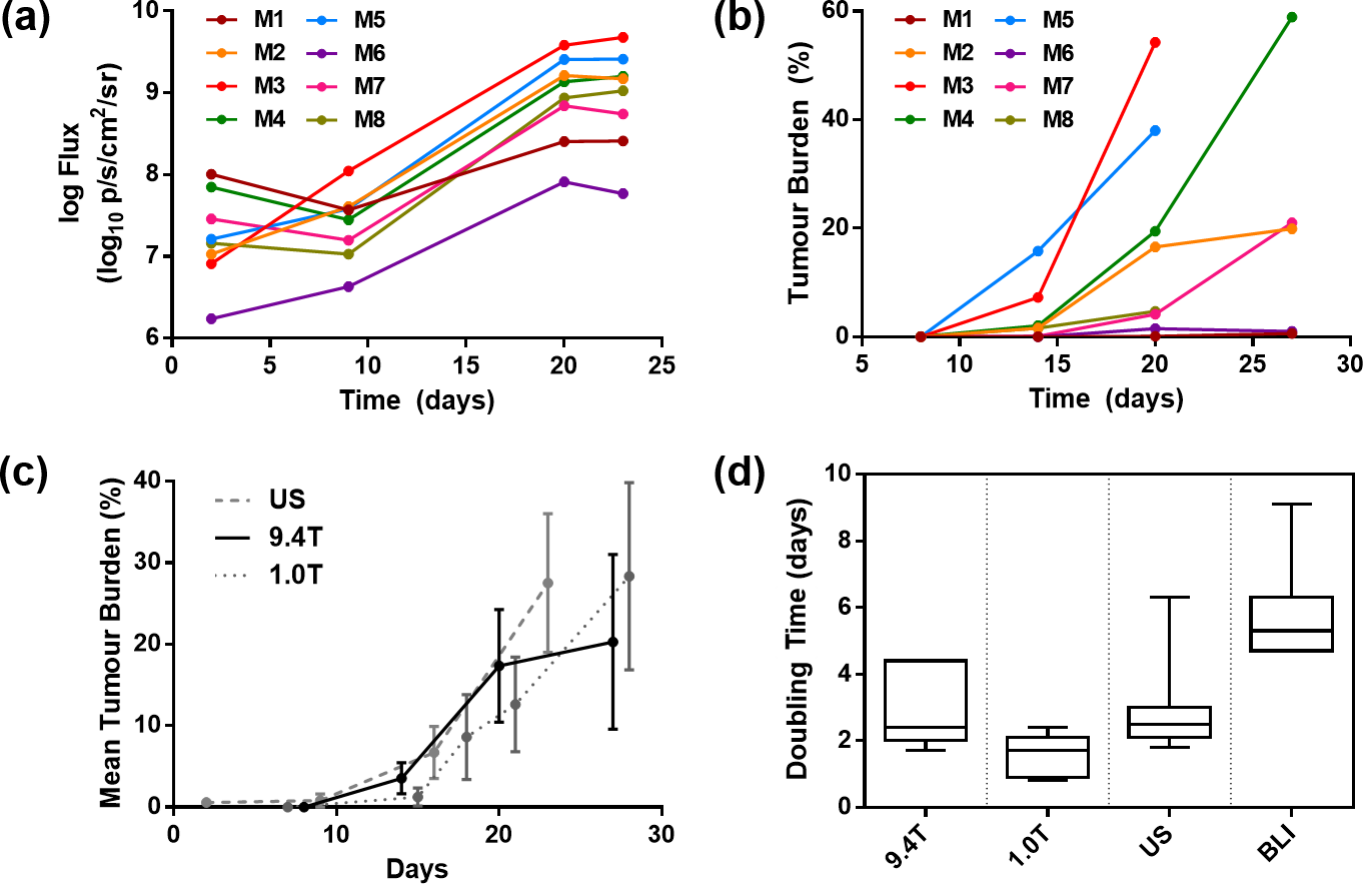
Multimodal assessment of tumour growth: (a) Individual tumour log-growth curves from BLI photon intensity across the whole study. Photon flux increased a hundred-fold between day 9 and day 20 and then plateaued. (b) Assessment of tumour burden, characterised by high-field MRI, in each mouse (M1, M2, M3, etc.). A large spread of tumour volumes was observed over the course of the study. (c) From group-wise plots (mean ± standard deviation) of tumour burden, a good agreement can be seen between US, high-field MRI and low-field MRI estimates of tumour burden. (d) Box-and-whisker plots representing measurements of tumour volume doubling times for the group, across all modalities. ANOVA analysis indicated no significant differences (p > 0.2) between the US, low- and high-field MRI estimates of tumour burden, but a significant overestimation (p < 0.01) for BLI.

The change in the log bioluminescence flux is shown in Fig 2A for all animals, in which the signal appears to plateau after 20 days. In four mice, signal intensity decreased at day 9, due to the presence of viable tumour cells at day 2 that did not immediately engraft. The individual growth curves of estimated tumour burden from high-field MRI data are shown in Fig 2B, which reveal substantial variability in tumour growth between animals. Mouse 1 and Mouse 6 exhibited a small tumour burden, which appears to plateau, which is also consistent with their associated low BLI flux.

For comparison, the mean growth curves for high-field MRI, low-field MRI and US are shown in Fig 2C. A close relation can be seen across the modalities; the apparent plateau of tumour burden in the high-field MRI is due to the removal of three animals with an excessively large tumour burden, prior to the final time point. This ‘plateau’ effect was not observed in 1T MRI data, as it underestimated the tumour burden prior to the removal of these mice, and the US imaging stopped before these mice were removed. No significant difference (p > 0.2, ANOVA) was measured between tumour volume doubling times (Fig 2D) estimated by high-field MRI (2.9 ± 1.1 days, mean ± standard deviation), US (3.0 ± 1.5 days) and low-field MRI (1.7 ± 0.6 days). The average doubling time estimated from BLI photon-flux was significantly longer, (5.8 ± 0.6 days, p > 0.01, ANOVA).

**Fig 3:**
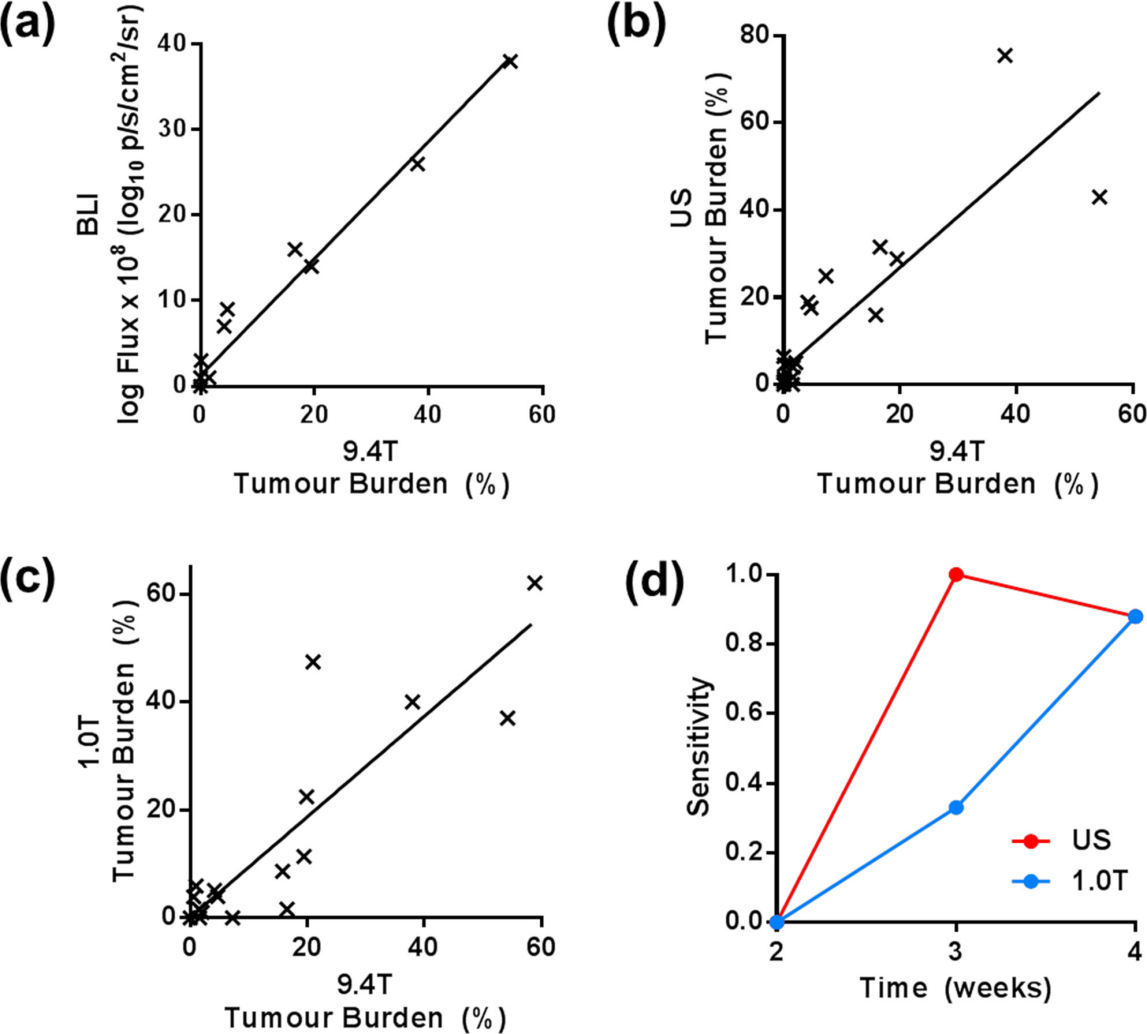
Correlation plots of tumour burden measured by high-field (9.4T) MRI (matched within three days) and each of the other modalities: (a) BLI r^2^ = 0.98, (b) US r^2^ = 0.84, (c) low-field (1T) MRI r^2^ = 0.89 (p < 0.001, Pearson’s, for all modalities). (d) The sensitivity of low-field MRI and US to detect tumours in the liver, using high-field MRI as a gold-standard.

Correlating BLI photon flux with high-field MRI estimates of tumour burden from the same time points (Fig 3A) showed a strong correlation (r^2^ = 0.98, p < 0.001, Pearson’s). Tumour burden measured by US (Fig 3B) was significantly correlated with estimates from high-field MRI (r^2^ = 0.84, p < 0.001, Pearson’s), as was tumour burden from low-field MRI (Fig 3C) (r^2^ = 0.89, p < 0.001, Pearson’s).

Measurements of the sensitivity of US and low-field MRI to detect tumours, compared to high-field (9.4T) MRI as a gold standard, revealed that both modalities were able to detect tumours by the week 3, though US was more sensitive at this time (Fig 3D). The two techniques were equally sensitive by week 4.

Table 1 shows a summary of the imaging parameters used for this study, and the results of the quantitative comparisons between the modalities. The experiment times are calculated from an approximate average time in minutes taken per mouse to set-up and acquire whole liver images. This was lowest for the BLI system, which has the potential to acquire data in five mice simultaneously, thereby offering a very high-throughput platform (4 minutes per mouse). The next fastest platform was US (10 minutes per mouse), followed by high-field MRI (20 minutes per mouse) and low-field MRI (40 minutes per mouse). The US procedure was shortened in this study with the use of nude mice, which did not require fur to be removed. The contrast-to-noise ratio (CNR) between tumour and liver tissue was greatest in high-field MRI data (4.9 ± 1.4, mean ± standard deviation), followed by low-field MRI (2.5 ± 0.9), and US (0.4 ± 0.1). Due to the specific nature of the BLI signal, the signal is only detected from tumour cells, thus contrast to liver tissue is not a comparable parameter, and hence not included in to Table 1. Placing a ROI distal from any mice at each week, a signal-to-noise of the BLI was estimated to be 1114 ± 947 (mean ± standard deviation); the large variance is due to the large spread of tumour uptake within the cohort, however the tumour signal was far greater than the background noise.

**Table 1:** Comparison of experimental parameters and derived properties across the modalities.

	Exp. Time (min)	Resolution (mm)	CNR	CV_U_ (%)	CV_A_ (%)	Doubling Time (days)
**BLI**	4	n/a	n/a	n/a	n/a	5.8 ± 0.6
**9.4T MRI**	20	0.15 x 0.15 x 0.75	4.9	4.7	7	2.9 ± 1.1
**1T MRI**	40	0.15 x 0.15 x 0.75	2.5	11.1	13.3	1.7 ± 0.6
**US**	10	0.018 x 0.018 x 0.076	0.4	14.3	9.9	3.0 ± 1.5

Experiment time (Exp. Time) is an approximate throughput time per mouse, including set-up and images acquisition, using the most efficient application of the protocols in this study. The image resolution in this table is that used in this study, and not the limit of the system. Contrast-to-noise ratio (CNR) was estimated using Eq 3, and describes the contrast between normal liver and tumour tissue in this study. The coefficients of intra-user (CV_U_) and inter-user (CV_A_) variability were calculated from Eq 2, and describe the reproducibility of tumour volume estimates, from image segmentation, for each modality. The final column shows the tumour volume doubling time (mean ± standard deviation) estimated from each modality.

Data acquired with high-field MRI, proposed as the gold-standard in this study, produced the smallest intra-user (CV_U_) and inter-user (CV_A_) variability during segmentation; these measures for low-field MRI and US segmentations were similar, most likely reflecting the trade-off between the CNR and resolution associated with each modality.

## Discussion

Pre-clinical, low-field (1T) MRI has not previously been assessed for its suitability to image liver tumour volumes, and was here evaluated alongside high-field (9.4T) MRI, ultrasound imaging (US) and bioluminescence (BLI). When comparing different technologies, a challenge exists to find a robust gold-standard to provide a set of data that is as close to a ground truth as possible. For the current study, in which the ability of several small animal imaging platforms to detect tumours and quantify their volume was evaluated in a mouse model of colorectal liver metastasis, high-field MRI was chosen as the gold-standard, and yielded the smallest variations in user assessment.

MRI signal increases with magnetic field, which is reflected in the reduced experimental time and greater CNR measured in this study with high-field MRI compared with low-field MRI. Though a good correlation was observed between the low-field and high-field MRI, the low-field MRI estimation of tumour doubling time was non-significantly shorter compared to the high-field MRI and US, suggesting a reduced sensitivity of the low-field MRI to the presence of smaller metastases. A non-significant, but consistent underestimation was observed in measurements of tumour burden by low-field MRI, largely due to the difficulty in distinguishing the inferior liver from the intestines with the acquired contrast: a 12% overestimation of liver volume was measured with the low-field MRI (data not shown), where the absolute tumour volumes were not significantly different, suggesting that measurements of absolute volume may be more suitable for the benchtop system.

The slice thickness of the US data was compressed by a factor of three to one, rather than matched to the slice thickness of the MRI data, in order to maintain the advantage of the fine step resolution offered by the 3D imaging. For a full quantification of tumour volume, manual segmentation is required, which can be time-consuming, although automated segmentation approaches can be developed for both this and MRI data [43]. However, though representative sampling of the US data covered approximately 25% of the whole liver volume, this method proved to be efficient and showed a strong correlation with tumour burden over the whole liver assessed by high-field MRI. More localised 3D imaging and whole volume analysis would be feasible for single-tumour models.

Individual BLI log flux growths exhibited a similar trend and produced a small variation in doubling time, suggestive that this technique is not sensitive to the variability in tumour burden. The significant overestimation in the doubling time measured in the BLI is likely to be due to the log flux signal plateau curve and may be better characterised by increasing the number of acquisitions earlier on in the study. In addition, a sigmoid function may be a better fit than a simple exponential, although this prevents straightforward comparison between modalities [44]. A limitation of the BLI in this study is that only one projection of the light emission was acquired, whereas a ‘tomographic’ approach may be more suitable to assess tumour burden and distribution [45]. Nonetheless, a strong correlation was observed between high-field MRI tumour burden estimates and BLI photon flux and could potentially be used to define a calibration curve for future studies, though this will require extensive characterisation for each model, cell line and system. Estimates of tumour burden, relative to normal liver, is likely to be closely linked to absolute tumour volume, which is demonstrated by the strong correlation between tumour burden from MRI and BLI signal, concurrent with previous studies [46].

The disadvantage of the BLI system is that it requires Luciferin to produce the light, whereas the other modalities used in this study did not require injected contrasts. The CNR of the MRI data could potentially be improved with the use of Gadolinium-based contrast agents, which may also shorten the acquisition time, though will require repeated intra-venous or intra-peritoneal administration.

A limitation of this study was that the sensitivity of the imaging modalities to measure tumour burden changes due to a therapy was not investigated. However, using the coefficients of variations reported here, future studies can be designed accordingly: to best appreciate the estimated coefficients of variation, they can be input into a two-sample, two-sided equality power calculation to predict each modalities utility in assessing tumour shrinkage following therapy. To measure a 10% change the high-field MRI, low-field MRI and ultrasound imaging will respectively require 4, 20 and 33 animals, using the intra-user CVs, a power of 80% and 5% type I error and assuming no significant contrast changes. Furthermore, studies that have measured on the variability of calliper estimations of tumour volume report CVs between 12–35%, and generally more varied than imaging such MRI, US and CT [5;22;35;47]. Thus calliper measurements, would mostly yield even larger required groups from power calculations than imaging.

A key parameter when evaluating the ability of each modality to detect tumours is the minimum tumour size that can be detected. This is primarily limited by the resolution of each of the imaging modality, alongside its signal-to-noise and contrast-to-noise characteristics. BLI was able to detect the presence of micro-metastases beyond the resolution of the MRI acquisition and sub-palpable volumes: viable cells within the liver at day 2, and successfully engrafted “micro-metastases” at day 9. For both MRI techniques used in this study, voxel volume was approximately 0.017 mm^3^, which is typical for preclinical MRI. This could be made smaller, but with the compromise of decreased signal-to-noise or increased imaging time, and the parameters used in this study aimed to provide a compromise between both of these factors. There is currently limited literature comparing US and MRI in application to pre-clinical tumour volumes, and a strong correlation was measured here between tumour burden estimations of high-field MRI and US. The results of the sensitivity analysis showed that US (pixel resolution approximately 7 x10^-5^ mm^3^) was more sensitive than low-field MRI. As such, US offers a particularly attractive, low-cost, and rapid platform for tumour burden assessment, even in the presence of artefacts such as rib shadowing, which may obscure tumours in the superior liver.

Although available, X-ray CT and nuclear imaging were not incorporated into this study. This was principally due to the limited number of scanning time points that could be performed, but also to avoid the use of modalities that require ionising radiation, particularly as there is some evidence that repeated exposure may impact on tumour growth [47].

An interesting observation was the variability found in doubling times associated with the growth of tumours in the liver, which, according to measurements with high-field MRI, varied between 1.7 and 4.4 days. This is consistent with results obtained from orthotopic liver metastasis models derived from other colorectal cancer cell lines [36;48;49], which reflect a more variable doubling time in orthotopic tumours than traditional ectopic models, also consistent with investigations of SW1222 metastases [50], though this does not report growth rates. In addition, a similar growth rate has been reported in subcutaneous models of SW1222 tumours; between 3 and 5 days [51;52], though xenograft models will typically grow to larger volumes. If a target tumour burden is defined as that at which a therapy study is to be initiated, this can introduce a significant source of variability, highlighting a key need for imaging when using orthotopic tumour xenograft models to evaluate novel therapies.

## Conclusion

Translational cancer research using orthotopic and transgenic models requires the non-invasive characterisation of tumour volume. Benchtop MRI has not been extensively reported in its utility, and here demonstrated that low-field MRI is an accurate, low cost method relative to high-field MRI and could offer straightforward application to tumour growth and potentially shrinkage measurements. Each of the comparison imaging modalities investigated in this study offer a complex array of advantages and challenges, thus it isn’t clear which technique is ‘best’ for assessing the presence of tumours and quantifying their volume. Such decisions will depend on the practical and scientific requirements of individual studies and will, we hope, be guided by the data presented in this study.

A combination of the applied modalities may provide the most complimentary data for assessing tumour models: BLI offers a highly specific and efficient method to screen for successful tumour engraftment and spread, where the other imaging modalities can then later be used for precise volumetric imaging. Such a workflow would be particularly advantageous for spontaneous transgenic models developed for optical imaging. A key observation from this study was the range of growth rates observed in the metastasis model was identified using each of the imaging modalities, suggesting that novel low field MRI and high-resolution US are suitable for characterising a range of liver tumour volumes.

## Supporting Information

S1 Fig Tiled bioluminescent image of all mice at all time points. (TIF)

S1 Table Tumour growth in burden (%) and flux density (p/s/cm^2^/str), for all mice by each modality. (XLSX)

S1 Video Video flythrough of an example raw data from 9.4T MRI, 1T MRI and ultrasound imaging of M5 at day 15. (MP4)
