# Supplementary figures and images for: Monitoring the Growth of an Orthotopic Tumour Xenograft Model: Multi-Modal Imaging Assessment with Benchtop MRI (1T), High-Field MRI (9.4T), Ultrasound and Bioluminescence
Source: PLoS One. 2016 May 25;11(5):e0156162. doi: 10.1371/journal.pone.0156162 (PMC4880291; doi:10.1371/journal.pone.0156162)

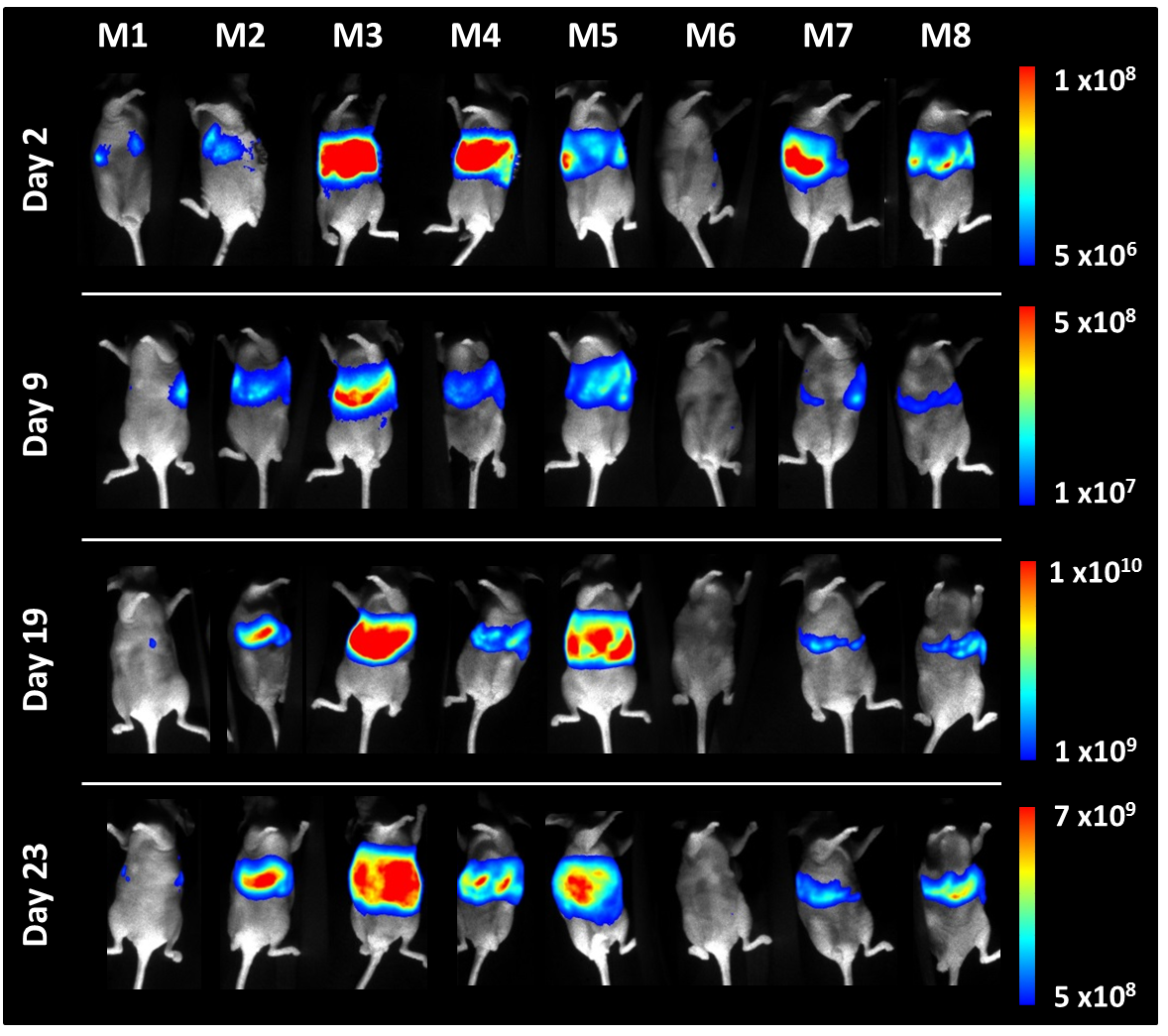

Supplement: S1 Fig — (TIF) [file pone.0156162.s001.tif]
